# Factor Structure of Urdu Version of the Flourishing Scale

**DOI:** 10.3389/fpsyg.2018.01513

**Published:** 2018-09-19

**Authors:** Fahad R. Choudhry, Yaser M. Al-Worafi, Bushra Akram, Mirza A. Ahmed, Muhammad Anwar ul Haq, Tahir Mehmood Khan, Inayat U. Rehman, Nadia Barki, Khadeeja Munawar, Anila Kamal, Yaman W. Kassab, Faizah S. Bakrin, Karen J. Golden

**Affiliations:** ^1^Department of Psychology, Jeffrey Cheah School of Medicine and Health Sciences, Monash University Malaysia, Bandar Sunway, Malaysia; ^2^National Institute of Psychology, Quaid-i-Azam University, Islamabad, Pakistan; ^3^Clinical Pharmacy Department, College of Pharmacy, Ajman University, Ajman, United Arab Emirates; ^4^Department of Psychology, University of Gujrat, Gujrat, Pakistan; ^5^Department of Management Sciences, University of Gujrat, Gujrat, Pakistan; ^6^Department of Pharmacy, Abasyn University, Peshawar, Pakistan; ^7^Institute of Pharmaceutical Science, University of Veterinary and Animal Sciences, Lahore, Pakistan; ^8^Department of Pharmacy, Abdul Wali Khan University, Mardan, Pakistan; ^9^Department of Psychology, University of Wah, Rawalpindi, Pakistan; ^10^Department of Hospital and Clinical Pharmacy, Cyberjaya University College of Medical Sciences, Cyberjaya, Malaysia; ^11^School of Pharmacy, KPJ Healthcare University College, Nilai, Malaysia

**Keywords:** psychometric properties, confirmatory factor analysis, mental well-being, psychological well-being, social psychological functioning, flourishing, Pakistan, positive psychology

## Abstract

**Background:** A great deal of research has been carried out on the assessment of the eudaimonic perspective of psychological well-being and the hedonic perspective of subjective well-being. The Flourishing Scale (FS) has been extensively used in research and practice, as it assesses the fundamental aspects of social psychological functioning. Nevertheless, the psychometric properties of Urdu versions of eudaimonic measures, such as the FS, have not yet been ascertained. The translation and validation of the FS in the Urdu language was not available, and hence this study was planned with the aim to validate the Urdu version of the FS.

**Methods:** We assessed the psychometric properties of the FS in a sample of adults aged 18 years and above in Pakistan (*N* = 130) using exploratory factor analysis based on principal component analysis with varimax rotation and confirmatory factor analysis.

**Results:** The exploratory factor analysis confirmed the unidimensional nature of the 8-item FS. We assessed that the Urdu version of the FS showed a high internal consistency reliability (α = 0.914) with a significant intraclass correlation coefficient (ICC), *p* < 0.001). In our study, the Kaiser–Mayer–Olkin value was 0.915 with a chi-square test value (χ^2^) of 637.687, and Bartlett's test of sphericity was significant (*df* = 28, *p* < 0.001). The intraclass correlation coefficients (ICCs) at test–retest for all domains were statistically significant (*p* < 0.001) and showed excellent agreement for all the items. The revised confirmatory factor analysis revealed a good-fit model, but with item 8—“People respect me”—removed due to its lower factor loading.

**Conclusions:** The findings suggest that the FS is a psychometrically sound instrument for assessing social psychological functioning among adults in Pakistan. Therefore, the validated Urdu version of the FS may be used in future studies of well-being in clinical psychology and positive psychology.

## Introduction

In recent decades, psychological well-being and subjective well-being have become a center of attention for researchers (Ryff, 1989; Diener et al., 1999; Diener and Biswas-Diener, 2008; Tong and Wang, 2017). Mental well-being, which encompasses both hedonic and eudaimonic perspectives on well-being, is considered a multifaceted construct that has undergone comprehensive and extensive empirical exploration (Schotanus-Dijkstra et al., 2016). The hedonic perspective refers to the emotional or “feeling good” dimension of well-being, and the eudaimonic perspective refers to psychological functioning or the “living well” dimension of well-being (Diener, 1984; Lee and Ishii-Kuntz, 1987). Subjective well-being is defined as comprising both affective and cognitive appraisals of one's quality of life (Diener, 1984; Tong and Wang, 2017). Because of a lack of consensus on the definition of subjective well-being, it has been the subject of ongoing empirical investigation (Bradburn, 1969; Diener, 1984; Diener et al., 1985, 2009; Hills and Argyle, 2002). Usually research studies based on the hedonic perspective of subjective well-being have used single-item or brief measures, such as the Satisfaction with Life Scale (Diener et al., 1985), the Subjective Happiness Scale (Lyubomirsky and Lepper, 1999), and the Positive and Negative Affectivity Scale (Watson et al., 1988). Despite the fact that these measures have undergone extensive research and practice, they have been unable to fully capture the social and psychological well-being dimensions (Schotanus-Dijkstra et al., 2016).

The eudaimonic perspective and its core aspects have recently been the focus of growing research attention (Schotanus-Dijkstra et al., 2016). Five core dimensions of social well-being were identified based on sociological and social psychological theories (Corey Lee, 1998). Likewise, six core dimensions of psychological well-being were identified based on an extensive review of humanistic, existential, and developmental theories (Ryff, 1989). All the social and psychological core aspects of well-being are present in the Mental Health Continuum-Short Form (MHC-SF), which also assesses hedonic well-being (Keyes, 2005; Keyes et al., 2008). Other comprehensive but generic measures for assessing well-being are the WHO-Five Well-being Index (Bech, 1999) and the Warwick–Edinburgh Mental Well-being Scale (Tennant et al., 2007).

Flourishing has been shown to be measured by the constructs of *meaning* and *achievement* as well as by an *affective component* (Bradburn, 1969; Diener, 1984; Watson et al., 1988; Seligman, 2011; Tong and Wang, 2017). According to research findings of an early study by Diener (1984), subjective well-being comprises life satisfaction and positive and negative affect. Other instruments developed by researchers to assess a variety of emotions and feelings include the Positive and Negative Affect Schedule (Watson et al., 1988), the Affect Balance Scale (Bradburn, 1969), and the Hedonic Balance Scale (Schimmack et al., 2002). There is a need for more exploratory empirical studies to better understand the concepts, definitions, and measurement of well-being (Busseri and Sadava, 2011; Tong and Wang, 2017). Relatedly, there is a need for such instruments that focus solely on the measurement of the core dimensions of the eudaimonic perspective (Schotanus-Dijkstra et al., 2016). Thus, a brief and comprehensive Flourishing Scale (FS) has recently been designed that is based on the humanistic and eudaimonic well-being perspectives (Diener et al., 2009; Schotanus-Dijkstra et al., 2016).

According to the majority of researchers, flourishing is a state in which high levels of subjective well-being as well as social psychological well-being are attained (Keyes, 2002; Seligman, 2011; Hone et al., 2013; Schotanus-Dijkstra et al., 2016). The first version of this scale had 12 items and was called the Psychological Flourishing Scale (Diener et al., 2008). It was followed by a revised and final version having eight items called the Psychological Well-being Scale (Diener et al., 2009). As this name was similar to Ryff's Scales of Psychological Well-being (Ryff, 1989), the Psychological Flourishing Scale was renamed as the FS (Diener et al., 2009). Owing to the briefness, easiness of use, and completeness of the FS, it has been used in a variety of well-being intervention studies and clinical practice and translated into 17 languages (Schotanus-Dijkstra et al., 2016). Across many samples, the psychometric properties of the FS have been established (Diener et al., 2009; Hone et al., 2013; Silva and Caetano, 2013; Howell and Buro, 2014; Sumi, 2014; Tang et al., 2016).

The psychometric properties of the FS have been assessed and various validated versions have been developed, such as versions in Portuguese, Japanese, Indian, Italian, Spanish, Croatian, Chinese, and in the language of the Netherlands (Silva and Caetano, 2013; Sumi, 2014; Duan and Xie, 2016; Schotanus-Dijkstra et al., 2016; Checa et al., 2017; Giuntoli et al., 2017; Ramírez-Maestre et al., 2017; Singh et al., 2017; Tong and Wang, 2017; Vujčić et al., 2017; Hojabrian et al., 2018).

Through confirmatory or exploratory factor analysis, research studies found the FS to have a single factor and found adequate to excellent Cronbach alpha reliability values ranging from 0.78 to 0.95 (Schotanus-Dijkstra et al., 2016). The majority of past validation studies have also supported the convergent validity of the FS (Checa et al., 2017; Giuntoli et al., 2017).

The translation of the FS (See Appendix) into the Urdu language and validation have not been carried out. Considering that Urdu is a national language of Pakistan, this validation study will help researchers use the FS in their studies within Pakistan or administer the FS to Urdu-speaking population in other parts of the world. According to the BBC (2014), Urdu is spoken around the world by approximately 100 million people. While it is a national language of Pakistan, it is also understood and spoken in parts of India, Bangladesh, the Middle East, Nepal, and other countries where Pakistani communities have settled.

## Materials and methods

Data for the present validation study were collected from January to April 2016 in Islamabad, the capital city of Pakistan. For recruiting participants, the study flyers were distributed in two academic institutes, four shopping malls, and six restaurants. No financial compensations were offered as this was not a funded project. Participants were informed that the collected data would be used only for research purposes, and they signed the written informed consent form. All participants agreed to voluntarily take part in the present study and completed the scale. Confidentiality of data was ensured, and anonymity of participants' identity was maintained.

### Participants

In the present study, a sample of adults in Pakistan aged 18 years and above (*N* = 130) was recruited. Of these, 111 were males (85.4%) and 19 were females (14.6%). Ages of participants ranged from 22 to 55 years (Median = 28.50 years, IQR = 24.75–32.00). Only those participants who were able to answer the statements of the scale in Urdu were recruited. Participants having any serious psychiatric illness (e.g., schizophrenia, bipolar disorder) or medical condition (e.g., cardiovascular problems, diabetes, cancer, etc.) were excluded. In terms of education, 13.85% (*n* = 18) had completed 6 years of education, 30.77% (*n* = 40) 12 years of education, 6.92% (*n* = 9) 15 years of education, 16.92% (*n* = 22) 16 years of education, and 31.54% (*n* = 41) 18 years of education. A majority of the participants-−67.69% (*n* = 88)—belonged to the Punjabi ethnic group of Pakistan.

### Sample size

Based on the ratio between the numbers of items in the scale to participants—that is 1:10—(Lai et al., 2013), the minimum sample size calculated was 80 participants. However, we recruited 130 participants for this study and at retest we had 119 participants.

### Instrument for translation

The FS was originally developed by Diener et al. (2009) and has eight dimensions that measured human functioning features. Previous studies have shown the adequate psychometric properties of the FS in various countries (Diener et al., 2010; Esch et al., 2013; Silva and Caetano, 2013; Hone et al., 2014; Sumi, 2014). For linguistic validation of the FS, the same eight dimensions were translated into Urdu according to the guidelines mentioned in the past studies (Guillemin et al., 1993; Wild et al., 2005).

To conduct forward translation, a panel of Urdu language experts (consisting of three persons) was invited for providing feedback regarding the exact translation of the English statements to Urdu. The purpose of inviting Urdu language experts was to help ensure that the tool could be easily understood by Urdu-speaking people. When this phase was completed, the instrument was given to a native Urdu language speaker for finalizing an initial version of the FS. To reassess the appropriateness of the translation, this translated questionnaire was given to the linguistic department. Linguistic experts suggested a few minor grammatical changes. After incorporating those suggestions, forward translation of the FS was achieved and backward translation was started (Wild et al., 2005). The Urdu version of the FS went through backward translation by experts. This scale was ready for face validity testing after backward translation was achieved.

The Urdu version of the FS was administered to 10 local Urdu-speaking Pakistanis as a pilot study for assessing face validity. The responses of these participants were not included in the final analysis of data. Necessary feedback and comments from the participants were noted down and the instrument was modified through mutual consensus. A practicing psychologist, with the qualification of Ph.D. in psychology and more than a decade experience of working in the field of psychology and research, performed content validity of the translated FS. The instrument was ready for further validity and reliability assessment after achieving content validity.

### Procedure

Participants were approached randomly in the locations mentioned earlier to assess the psychological well-being of the general population and the scale items were presented in random order. Furthermore, there were no significant differences in the FS mean score between participants who completed the scale at the initial stage of the study and those who did so at the end of the study. To assess the reliability, the Urdu FS was administered to the same participants 4 weeks later. The administration of scale took <15 min.

### Statistical analysis

To conduct the analysis, we used SPSS version 20.0 (SPSS Inc., Chicago, IL). Normality was assessed using the Shapiro–Wilks test. Non-parametric tests were performed as the data was not normally distributed. To present the continuous and categorical variables, median and interquartile range, as well as number and frequency were used, respectively. Exploratory factor analysis was performed to examine the construct validity of the FS. The eigenvalues were retained for a factor >1 because of their significant contribution in explaining the overall model variation. Furthermore, to assess the sampling adequacy, the Bartlett's test of sphericity and the Kaiser–Mayer–Olkin (KMO) were used. KMO values below 0.5 are considered “unacceptable” (Kaiser, 1974); values that fall in the range of 0.5–0.7 are considered “mediocre;” “good” values lie in the range of 0.7–0.8; the range of 0.8–0.9 gives “great” values, while values >0.9 are considered “superb” (Field, 2009). The Mann–Whitney *U*-test was conducted to determine the discriminative validity, between baseline and retest as well as between males and females from the same sample. Cronbach's alpha was used for examining internal consistency. Values >0.9 are graded as excellent, >0.8 as good, more than 0.70 as acceptable, and >0.6 as questionable (George, 2003). Additionally, the intraclass correlation coefficient (ICC) was used to examine the reliability. ICC values >0.75 indicate excellent agreement, in the range 0.60–0.74 indicate good agreement, in the range 0.40–0.59 show fair to moderate agreement, and <0.40 indicate poor agreement (Hutcheson and Sofroniou, 1999; McDowell, 2006).

## Results

A total of 130 participants were recruited for the present study out of which 85.4% were males. The median age of the participant was 28.50 years with an IQR [24.75–32.00]. Approximately 31.54% of participants reported having a postgraduate education [18 years of education], followed by 30.77% of participants having a secondary level education [12 years of education]. Among the participants, 67.69% were Punjabi, and 20.77% were Pashtoon, as shown in Table 1. However, all the ethnic groups do understand and speak Urdu as it is the national language of Pakistan.

**Table 1:** Demographic characteristics of participants of the FS (*N* = 130).

**Demographics**	***N* (%)**
**GENDER**	
Male	111 (85.4)
Female	19 (14.6)
**AGE (YEARS)**	
Median	28.50
[IQR]	[24.75–32.00]
**EDUCATION**	
Primary level [6 years of education]	18 (13.85)
Secondary level [12 years of education]	40 (30.77)
College/Diploma [15 years of education]	9 (6.92)
Undergraduate [16 years of education]	22 (16.92)
Postgraduate [18 years of education]	41 (31.54)
**ETHNICITY**	
Punjabi	88 (67.69)
Pashtoon	27 (20.77)
Sindhi	12 (9.23)
Gilgit/Skardu	1 (0.77)
Others	2 (1.53)
**PERCEIVED SOCIOECONOMIC STATUS**	
Low	14 (10.77)
Lower-Middle	27 (20.77)
Middle	51 (39.23)
Middle-High	33 (25.38)
High	5 (3.84)

### Construct validity (factor analysis)

#### Confirmatory factor analysis (CFA)

CFA was performed to validate the proposed model. Overall, the goodness of fit was evaluated by examining numerous fit indices. The fit indices used to examine this model were the chi square (χ^2^), comparative fit index (CFI), p of close fit (PCLOSE), and root-mean-square error of approximation (RMSEA). Browne and Cudeck (1993) specify the cut-off criteria for CFI as >0.90. For relative χ^2^, cut-off criteria vary from a higher value of 5.0 (Wheaton et al., 1977) to a lower value of 2.0 (Tabachnick and Fidell, 2007). For RMSEA, the cut-off lies at <0.06–0.08 (Schreiber et al., 2006).

**Figure 1:**
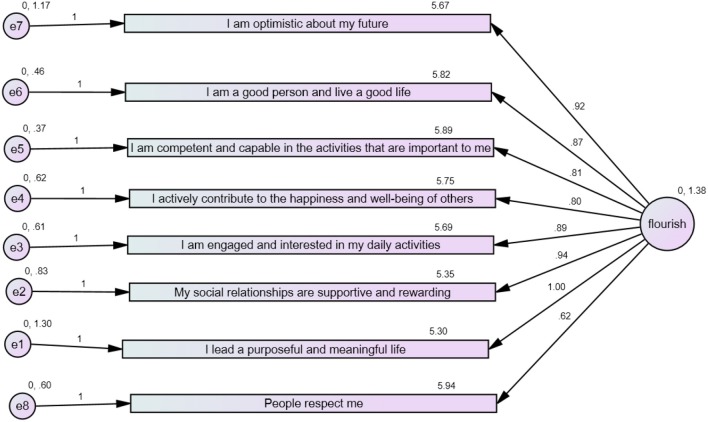
Factor loading of all items.

CFA for the FS scale was carried out with all eight items (Figure 1). The analysis revealed that the model came out as bad fit as indices indicated: χ^2^ = 2.34, *p* = 0.001, CFI = 0.95, RMSEA = 0.102, and PCLOSE = 0.01. Factor loadings revealed that item 8—“People respect me”—had a factor loading of 0.62, which is comparatively lower than other factor loadings. A lower factor loading on this item indicates that item 8 does not represent the latent variable “flourishing” fully. Therefore, this was considered to be the reason of a bad-fit model. No indices were modified for the initial run. However, to improve the model and retain item 8, indices were modified, but the model revealed similar results as explained above. Figure 1 shows the model fit with item 8.

**Figure 2:**
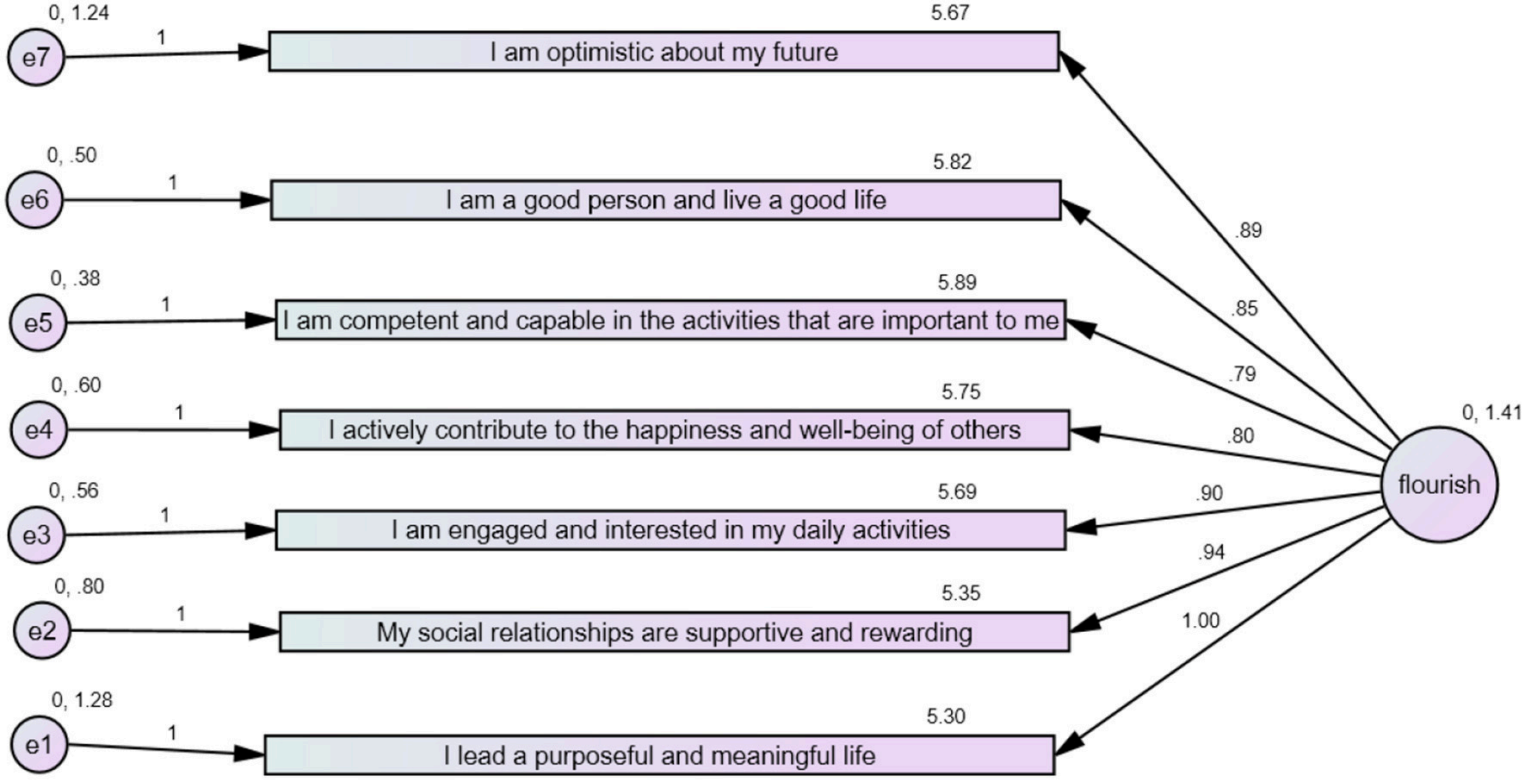
Factor loading of all items except item 8.

To improve the model fit, CFA was performed again (Figure 2) but with item 8 removed owing to its lower loading. The analysis now revealed a good-fit model as indicated by indices:

χ^2^ = 1.64, *p* = 0.06, CFI = 0.98, RMSEA = 0.07, and PCLOSE = 0.23.

The CFA indicated that the original model could not be confirmed and modifications had to be made to obtain a good-fit model.

#### Exploratory factor analysis

This analysis revealed that the Urdu FS had 1-factor loadings based on principal component analysis with varimax rotation, this shows that with 64.037% of the variance [KMO = 0.915, chi-square = 637.687, Bartlett's test of sphericity was significant (*df* = 28, *p* < 0.001)].

The Mann–Whitney *U*-test was used to determine whether the Urdu FS was able to discriminate between males and females from the same sample. The significance level was set at *p* < 0.05. The overall score of the FS, as well as for each domain, was somewhat better for female participants (47.10 ± 6.01) compared to males (45.12 ± 8.78). However, the differences were not statistically significant (Table 2).

**Table 2:** Scores of the Urdu FS among male and females.

**Domains**	**Males**			**Females**			**Mann–Whitney** ***U***-**test**	
	**Median**	**[IQR]**	**Mean ± SD**	**Median**	**IQR**	**Mean ± SD**	**z-score**	***p*-value**
Purpose and meaning	6.00	[2.00]	5.31 ± 1.66	6.00	[2.00]	5.26 ± 1.59	−0.173	0.86
Supportive relationships	6.00	[1.00]	5.28 ± 1.46	6.00	[2.00]	5.79 ± 1.22	−1.588	0.11
Engagement	6.00	[2.00]	5.65 ± 1.32	6.00	[2.00]	5.95 ± 1.22	−1.041	0.29
Contribution to the well-being of others	6.00	[2.00]	5.72 ± 1.27	6.00	[2.00]	5.89 ± 0.93	−0.248	0.81
Competence	6.00	[2.00]	5.87 ± 1.18	6.00	[0.00]	6.00 ± 0.74	−0.095	0.92
Self-acceptance	6.00	[2.00]	5.80 ± 1.27	6.00	[2.00]	5.95 ± 0.97	−0.163	0.87
Optimism	6.00	[2.00]	5.59 ± 1.62	6.00	[1.00]	6.11 ± 0.89	−0.798	0.42
Being respected	6.00	[1.00]	5.90 ± 1.12	6.00	[1.00]	6.16 ± 0.68	−0.564	0.57
Overall FS	47.00	[9.00]	45.12 ± 8.78	46.00	[7.00]	47.10 ± 6.01	−0.528	0.59

*IQR, interquartile range*.

Discriminative validity was assessed using the Mann–Whitney *U*-test to determine if there was no significant difference between test–retest reliability scores. The median scores for the FS for all the domains at baseline (*n* = 130) and retest (*n* = 119) are shown in the table below. The table shows similar interpretation of responses by participants at baseline and retest. The scale is therefore stable, as shown in Table 3. The Mann–Whitney *U*-test was used in this study because the aim was to compare two sample means of the same population (i.e., males and females and test–retest reliability scores).

**Table 3:** Scores of the participants at the test (baseline) and retest of the Urdu FS.

**Domains**	**Test** ***(n*** = ***130)***		**Retest** ***(n*** = ***119)***		**Mann–Whitney *U*-test**
	**Median**	**IQR**	**Median**	**[IQR]**	***p*-value**
Purpose and meaning	6.00	[2.00]	6.00	[1.00]	0.99
Supportive relationships	6.00	[1.00]	6.00	[2.00]	0.2
Engagement	6.00	[2.00]	6.00	[2.00]	0.99
Contribution to the well-being of others	6.00	[2.00]	6.00	[2.00]	0.97
Competence	6.00	[1.00]	6.00	[2.00]	0.13
Self-acceptance	6.00	[2.00]	6.00	[2.00]	0.68
Optimism	6.00	[2.00]	6.00	[2.00]	0.85
Being respected	6.00	[1.00]	6.00	[1.00]	0.70
Overall FS	47.00	[9.00]	47.00	[10.00]	0.88

*IQR, interquartile range*.

The overall Cronbach's alpha for the Urdu FS was 0.914. For retest, 11 participants did not show up owing to personal reasons and busy schedules, and they were dropped for the follow-up. The ICC at test–retest for all domains were statistically significant (*p* < 0.001) and showed excellent agreement for all items (Table 4).

**Table 4:** The psychometric properties of the FS.

**Domain**	**Cronbach's alpha for overall instrument**	**Corrected item total correlation**	**Cronbach alpha if item deleted**	**Test (*n* = 130)**	**Retest (*n* = 119)**	Intraclass correlation coefficient (ICC)^*^
				**Median**	**Median**	
Purpose and meaning	0.914	0.682	0.909	6.00	6.00	0.99^*^
Supportive relationships		0.739	0.901	6.00	6.00	0.61^*^
Engagement		0.770	0.899	6.00	6.00	0.98^*^
Contribution to the well-being of others		0.724	0.903	6.00	6.00	0.99^*^
Competence		0.794	0.898	6.00	6.00	0.40^*^
Self-acceptance		0.793	0.897	6.00	6.00	0.97^*^
Optimism		0.671	0.908	6.00	6.00	0.99^*^
Being respected		0.661	0.908	6.00	6.00	0.99^*^

* *Statistically significant at p < 0.05*.

## Discussion

This is the first study to evaluate the construct validity and reliability of the Flourishing Scale in Urdu in a sample of 130 adults. Findings from the exploratory analysis support the unidimensionality of this scale. Our study also examined the retest reliability at a time interval of 1 month, similar to the study carried out by Diener et al. in 1999 (Keyes et al., 2008).

Like the previous versions of the FS (Diener et al., 2010; Hone et al., 2013; Silva and Caetano, 2013), the Urdu FS has shown adequate reliability (Henson, 2001). The present research study also helps fill the knowledge and research gaps and would ease future research studies on samples understanding the Urdu language. Findings of the current study are in harmony with the results of past research (Ryff, 1989; Diener and Biswas-Diener, 2008; Seligman, 2011). This study has added evidence pertaining to the dimensionality and assessment of flourishing.

However, of note, in CFA, an item designed to measure “social relationships” (i.e., item # 8: “*People respect me*”) as a component of flourishing was supported to be discarded. The conceptualization of flourishing by Keyes (2007), Forgeard et al. (2011), and Huppert and So (2013) do not consider the observed variable “social relationships” through the respect from others in the operationalization of the latent variable “flourishing.” Therefore, it can be assumed that weak theoretical justification may be the reason for the lower factor loading on item 8 (i.e., “People respect me”), which resulted in the removal of this item to improve the goodness of fit. Furthermore, the cultural difference between the current Pakistani sample and the original sample may have played a role in the lower factor loadings. Variation in conceptualization can induce differences in the prevalence of a construct (Hone et al., 2014) across cultures. Therefore, it can be argued that some items may not fully represent the said construct in a culture, resulting in the suggested removal of those items. Further research is strongly suggested to explore about this possibility.

Regarding the presence of gender differences in the FS, mixed evidence exists. Some studies have shown the presence of gender differences (Howell and Buro, 2014), whereas others have shown the absence of any differences due to gender (Diener et al., 2010). In harmony with the study conducted by Diener et al. (2010), our study also did not find any significant gender differences (Diener et al., 2010), although some caution is recommended when interpreting this result considering the significantly smaller number of females than males in the current sample. Small but non-significant gender differences in our study can be supported by the study of Arrosa and Gandelman (2016). According to Arrosa and Gandelman (2016), all around the world, females are found to be happier (Arrosa and Gandelman, 2016).

Although the tool was translated into Urdu in some previous research studies, it was uncertain whether such studies have established the psychometric properties of the Urdu version of the FS. Thus, the current study helped fill the research gap and presented some evidence regarding the application of the FS (Diener et al., 2010) in a sample of Pakistani adults and may permit the findings to be better generalized to Pakistani people from varying backgrounds. This could help facilitate the future comparison of well-being of people from social groups with different ages and social status. Greater cross-cultural studies of eudaimonic well-being would be beneficial in the literature and could help facilitate the conceptual understanding of this complex construct.

In harmony with the original English version of the FS (Diener et al., 2010), we found that the Urdu version of the FS showed a high internal consistency reliability (α = 0.914) with a significant ICC (*p* < 0.001) (Nunnally and Bernstein, 1994; Henson, 2001). In our study, the KMO value was 0.915 with a chi-square value χ^2^ of 637.687, and the Bartlett's test of sphericity was significant (*df* = 28, *p* < 0.001). The ICC at test–retest for all domains was statistically significant (*p* < 0.001) and showed excellent agreement for all items. As the English version of the FS did not assess the ICC, we were not able to compare our ICC results with that of the previous studies. In addition, the discriminative validity established no significant changes in test (baseline) and retest results. We were not able to compare our results with the results obtained with the English version of the FS, as discriminative validity was not assessed.

In a nutshell, it can be said that the aim of this study was achieved and results have demonstrated that the Urdu version of the FS has good psychometric properties resembling those verified in the original study (Diener et al., 2010). This version of the FS has also shown excellent reliability and good factorial validity. The CFA revealed the good-fit model for seven items and one item (Item 8) was discarded. However, in future studies, the psychometric properties of the Urdu version of the FS should be assessed in other cohorts, for instance adolescents. In addition, the Urdu FS should be correlated with other instruments and scales of subjective and psychological well-being, such as the Personal Wellbeing Index (Group, 2006), the Basic Needs Satisfaction Scale (Ryan and Deci, 2001), and the Ryff's scales of Psychological Well-being (Ryff and Keyes, 1995).

## Limitations

The strength of our study is that this tool was translated and validated in Urdu to access flourishing/well-being in the Pakistani population, addressing a gap in the literature. One limitation of the study was geographical, as the data were collected from only one city due to convenience sampling. Another limitation of this study was the non-balanced gender sample, as only a small sample of females was recruited; a more balanced gender sample would have given more representative findings. In addition, our sample in this study was based on a higher educated population than the mean in the country. Furthermore, considering the cross-sectional design of this study, a small sample size, and the limited measure available to assess the aspects of concurrent validity, further studies on larger and gender-balanced samples are required for a greater depth of validation of the Urdu version of the FS.

## Conclusion

The Urdu version FS is suggested as a valid and reliable measure for assessing subjective well-being among the Pakistani population. The validated Urdu FS tool can be used in clinical psychology and positive psychology studies to access well-being. However, future research is strongly recommended to continue to assess the scale reliability and validity in more subpopulations and in more depth.

## Ethics statement

This study was approved by the ethics committee of the Abasyn University in Peshawar, Pakistan.

## Author contributions

FRC and KM collected and analyzed the data and prepared the manuscript. KG supervised the whole process and approved the final version of the manuscript to be published and also helped in proofreading and providing technical psychological insights. NB, AK, and YA-W helped in revising the manuscript and contributed into the result and method sections and also helped in performing the statistical analysis. TMK, IUR, FRC, KM, BA, MAA, MA, FB, and YK helped in performing the statistical analysis, revising the analysis and checking and drafting of the results section. KG, NB, AK, YA-W, BA, MAA, MA, FB, and YK helped in running the Confirmatory Factor Analysis, checked the Exploratory Factor Analysis and interpreted the data. Moreover, they also played a significant role in revising the manuscript after peer review. All the current and new authors approved the final version of the manuscript to be published.

### Conflict of interest statement

The authors declare that the research was conducted in the absence of any commercial or financial relationships that could be construed as a potential conflict of interest.

## Appendix

English FS

**Table:** Below are 8 statements with which you may agree or disagree. Using the 1–7 scale below, indicate your agreement with each item by indicating that response for each statement. Kindly place a check mark in the appropriate boxes:

	**Strongly disagree**	**Disagree**	**Slightly disagree**	**Neither agree nor disagree**	**Slightly agree**	**Agree**	**Strongly agree**
1. I lead a purposeful and meaningful life.							
2. My social relationships are supportive and rewarding.							
3. I am engaged and interested in my daily activities.							
4. I actively contribute to the happiness and well-being of others.							
5. I am competent and capable in the activities that are important to me.							
6. I am a good person and live a good life.							
7. I am optimistic about my future.							
8. People respect me.							

Urdu Version of FS
